# Comparison of two methods to report potentially avoidable hospitalizations in France in 2012: a cross-sectional study

**DOI:** 10.1186/s12913-014-0661-7

**Published:** 2015-01-22

**Authors:** Rodolphe Bourret, Grégoire Mercier, Jacques Mercier, Olivier Jonquet, Jean-Emmanuel De La Coussaye, Philippe J Bousquet, Jean-Marie Robine, Jean Bousquet

**Affiliations:** Centre Hospitalier Universitaire, Montpellier, France; MACVIA-LR: Fighting Chronic Diseases for Active and Healthy Ageing (Reference Site of the European Innovation Partnership on Active and Healthy Ageing), Montpellier, France; University of Montpellier 1, Montpellier, France; Centre Hospitalier Universitaire, Nîmes, France; Inserm, U710 and 988, Montpellier, France

**Keywords:** Diagnosis-related groups, International classification of disease, Potentially avoidable hospitalizations, PMSI, France

## Abstract

**Background:**

Potentially avoidable hospitalizations represent an indirect measure of access to effective primary care. However many approaches have been proposed to measure them and results may differ considerably. This work aimed at examining the agreement between the Weissman and Ansari approaches in order to measure potentially avoidable hospitalizations in France.

**Methods:**

Based on the 2012 French national hospital discharge database (*Programme de Médicalisation des Systèmes d’Information*), potentially avoidable hospitalizations were measured using two approaches proposed by Weissman et al. and by Ansari et al. Age- and sex-standardised rates were calculated in each department. The two approaches were compared for diagnosis groups, type of stay, severity, age, sex, and length of stay.

**Results:**

The number and age-standardised rate of potentially avoidable hospitalizations estimated by the Weissman et al. and Ansari et al. approaches were 742,474 (13.3 cases per 1,000 inhabitants) and 510,206 (9.0 cases per 1,000 inhabitants), respectively. There are significant differences by conditions groups, age, length of stay, severity level, and proportion of medical stays between the Weissman and Ansari methods.

**Conclusions:**

Regarding potentially avoidable hospitalizations in France in 2012, the agreement between the Weissman and Ansari approaches is poor. The method used to measure potentially avoidable hospitalizations is critical, and might influence the assessment of accessibility and performance of primary care.

## Background

The concept of potentially avoidable hospitalizations (PAH) or ambulatory care sensitive hospitalizations (ACSH) was proposed as an indirect measure of access to effective primary care [1-5]. It has also been used as a marker of overall healthcare system performance [6,7]. This approach is appealing since a large number of states, regions or hospitals have reliable data on hospital discharges and limited data on ambulatory care [8]. Avoiding admission represents a substantial reduction in costs, enhances patients’ quality of life and is an issue of considerable interest to policy makers and the public [9]. Higher rates of PAH are associated with socioeconomic deprivation [10,11], poor accessibility to primary care [5], and deficient continuity of care [12]. PAH can be reduced by programs aimed at improving primary care delivery [13,14] or by multifaceted interventions [15]. Thus PAH rates might increase where access to GPs is lower and where primary care is suboptimal.

In France, primary care is mostly delivered by self-employed physicians in the ambulatory care sector. General Practitioners have been playing a semi gatekeeping role since the late 1990s. Although patients are financially incentivized to visit their GP before being referred to a specialist, the gate-keeping procedure is not mandatory and patients can visit any specialist. A major weakness lies in the lack of coordination and continuity of care between GPs, ambulatory care and hospital care [16]. So far, GPs’ individual performance measures do not include PAH rates.

There are significant disparities for PAH by ethnicity, insurance status, and socioeconomic status [17]. PAH are common in older people [18-20] but can occur at any age [21-24]. Chronic diseases represent an important cause of PAH [3]. Comorbidities are associated with PAH [25].

Measuring PAH is important for policymakers and researchers willing to assess the performance of the primary care sector and to monitor the impact of interventions aimed at improving access. Trends in PAH are available for many countries [26-30] including France [31,32]. The estimation of the proportion of PAH among all hospital discharges is based on the assumption that hospitalizations for some conditions are preventable. Each of the conditions is defined by a list of diagnosis codes from the International Classification of Diseases (ICD). Two of the most critical issues include the selection of categories of conditions (diseases or complications of diseases) and of the corresponding ICD codes. Therefore, different methods have been used, that vary in terms of conditions and ICD codes. In France, Gusmano et al. [31,32] used the Weissman et al. approach [4], but this method does not encompass all potentially avoidable conditions. As an example, status asthmaticus, the most severe PAH in asthma, is not coded (ICD10 code J46), whereas it is coded in other studies [23]. Moreover, COPD (J20, J41, J42, J43, J44, J47 [23]), the most common cause of death due to respiratory diseases [33,34] and a common cause of PAH [5], is not even considered by Weissman et al. [4].

The current study aimed at estimating PAH in France in 2012 using the French hospital discharge database (*Programme de Médicalisation des Systèmes d’Information*; PMSI). Firstly, the Weissman et al. approach [4], already tested in France, was compared with the more recent Ansari one [23] at the French national and departmental levels. ICD-10 codes were used exactly as proposed by the authors.

## Methods

### Study population

The data for all patients hospitalized in France in 2012 were collected from the national administrative database, the PMSI. French public and private hospitals are financed through a Diagnosis-Related Group (DRG)-based prospective payment system [35,36]. The PMSI is the national discharge database and includes all hospital discharges from all public and private hospitals in France. The quality of this database is deemed well, especially since 2007 [37]. It centralises data by a diagnosis that is encoded according to ICD-10, medical procedure, age, residence and French diagnosis-related groups of patients admitted to all hospitals (public and private). The reliability and validity of the PMSI database have already been demonstrated for various acute and chronic conditions [38-41]. Hospital discharges are classified in diagnosis groups (*catégories majeures diagnostiques*, CMD) and then in DRGs according to ICD-10 principal and secondary diagnosis codes, surgical and non-surgical procedures (*Classification Commune des Actes Medicaux*, CCAM) [42] and age. We extracted discharge data for acute hospital stays in medicine, surgery and obstetrics/gynaecology. Discharges for foreign patients were excluded.

This research was approved by the Commission Nationale Informatique et Liberte, an independent ethic committee (www.cnil.fr; approval number DE-2013-118). Access to the PMSI database is free for researchers after approval by the CNIL. Written informed consent from patients is not required by French law for such studies.

### PAH definition

Since there is no consensus on the best one, two methods were independently used to identify PAH among all hospital discharges. Weissman et al. [4] (Table 1) was initially used since this is the standard approach for French studies [31,32]. In his seminal paper, Weissman used ICD-9 codes (Weissman 1992). We have used both the original method [4] and the ICD-10 conversion published by Gusmano et al. [43]. Only principal diagnosis codes were considered. However, the list of categories is not in accordance with the latest review carried out by Rosano et al. [5]. There are important missing diseases (e.g. Chronic Obstructive Pulmonary Diseases, COPD) and the codes reported may not be in full conformity with the current ICD-10 classification. We therefore used a second approach recently published by Ansari et al. used to assess and monitor access to primary care in Victoria, Australia [23] (Table 2). The strength of this method is that it encompasses a broader range of conditions, including COPD.

**Table 1:** **PAH selection algorithm according to Weissman et al. modified by Gusmano et al.**

**Category**	**ICD-10 codes (Principal diagnosis only)**
Bacterial pneumonia	J13, J14, J15, J16.0, J16.8, J18
Congestive heart failure	I50
Asthma	J45
Cellulitis	J34.0, K12.2, L02, L03
Complications of peptic ulcer disease	K25.0, K25.1, K25.2, K25.4, K25.5, K25.6, K26.0, K26.1, K26.2, K26.4, K26.5, K26.6, K27.0, K27.1, K27.2, K27.4, K27.5, K27.6, K28.0, K28.1, K28.2, K28.4, K28.5, K28.6
Pyelonephritis	N10, N11, N12, N13.6, N15.8, N15.9, N17.2
Type 2 diabetes mellitus with hyperosmolarity or coma	E10.0, E10.1, E11.0, E11.1, E13.0, E13.1, E14.0, E14.1
Ruptured appendix	K35.2, K35.3
Hypertension	I10, I11.0, I11.9, I12.0, I12.9, I13.0, I13.1, I13.2, I13.9, I15.0, I15.1, I15.2, I15.8, I15.9, I67.4
Hypokalaemia	E87.6
Immunizable conditions	A35, A36, A37, A80, B05, B26
Gangrene	I73.0, L88, I70.2

**Table 2:** **PAH selection algorithm according to Ansari et al.**

**Category**	**ICD-10 codes**	**Notes**
Influenza and pneumonia	J10, J11, J13, J14, J15.3, J15.4, J15.7, J15.9, J16.8, J18.1, J18.8	In any diagnosis field, exclude cases with secondary diagnosis of D57, and people under 2 months
Other vaccine preventable	A35, A36, A37, A80, B05, B06, B16.1, B16.9, B18.0, B18.1, B26, G00.0, M01.4	In any diagnosis field
Asthma	J45, J46	Principal diagnosis only
Congestive heart failure	I50, I11.0, J81	Principal diagnosis only, exclude cases with procedure codes
Diabetes complications	E10.1, E10.2, E10.3, E10.4, E10.5, E10.6, E10.7, E10.8, E11.0, E11.1, E11.2, E11.3, E11.4, E11.5, E11.6, E11.7, E11.8, E13.0, E13.1, E13.2, E13.3, E13.4, E13.5, E13.6, E13.7, E13.8, E14.0, E14.1, E14.2, E14.3, E14.4, E14.5, E14.6, E14.7, E14.8	In any diagnosis field
Chronic obstructive pulmonary disease	J20, J41, J42, J43, J44, J47	Principal diagnosis only, J20 only with diag2 of J41 J42 J43 J47 J44
Angina	I20, I24.0, I24.8, I24.9	Principal diagnosis only, exclude cases with procedure codes
Iron deficiency anaemia	D50.1, D50.8, D50.9	Principal diagnosis only
Hypertension	I10, I11.9	Principal diagnosis only, exclude cases with procedure codes
Nutritional deficiencies	E40, E41, E42, E43, E55.0, E64.3	Principal diagnosis only
Dehydration and gastroenteritis	E86, K52.2, K52.8, K52.9	Principal diagnosis only
Pyelonephritis	N39.0, N10, N12, N11, N13.6	Principal diagnosis only
Perforated/ bleeding ulcer	K25.0, K25.1, K25.2, K25.4, K25.5, K25.6, K26.0, K26.1, K26.2, K26.4, K26.5, K26.6, K27.0, K27.1, K27.2, K27.4, K27.5, K27.6, K28.0, K28.1, K28.2, K28.4, K28.5, K28.6	Principal diagnosis only
Cellulitis	L03, L04, L08, L98.0, L88, L98.3	Principal diagnosis only, exclude cases with procedure codes
Pelvic inflammatory disease	N70, N73, N74	Principal diagnosis only
Ear, nose and throat infections	H66, H67, J02, J03, J06, J31.2	Principal diagnosis only
Dental conditions	K02, K03, K04, K05, K06, K08, K09.8, K09.9, K12, K13	Principal diagnosis only
Convulsions and epilepsy	O15, G40, G41, R56	Principal diagnosis only
Gangrene	R02	Principal diagnosis only

For each method, any hospitalization with at least one of the ICD codes was systematically identified as potentially avoidable.

Hospitalizations were described using diagnosis-related groups of patients (CMD) from the French DRG system based on diagnosis codes (ICD-10) and surgical procedure codes (CCAM). The CMDs represent the first step of the classification algorithm. They are based on ICD-10 principal diagnosis codes [36].

Severity of disease was based on comorbidities, complications and age according to the French DRG grouping system. Four severity levels are defined from 1 (lowest severity) to 4 (highest). However, severity levels do not exist for all DRGs and we used those proposed.

Sex, age and length of stay are mandatory data for each discharge in the PMSI database.

### Statistical analysis

PAH were identified according to the Weissman et al. [4], Weissman modified by Gusmano et al. [43] and Ansari et al. approaches [23]. In 2012, they were identified for the 98 French departments overall and for the most frequent diagnosis groups. The proportion of PAH was calculated by dividing the number of PAH by the total number of hospital discharges in 2012. PAH crude rates were calculated by dividing the number of PAH by the 2011 national and departmental populations. Age- and sex-standardised rates of PAH were calculated in each department, employing the direct standardization method using the 2011 French population (*Institut National de la statistique et des etudes économiques - National Institute for statistics and economic studies*: http://www.insee.fr/fr/bases-dedonnees/default.asp?page=recensement/resultats/2011/donnees-detaillees-recensement-2011.htm (accessed on 20th Sept 2013, INSEE)). The agreement between both approaches was assessed graphically. The two approaches were compared for diagnosis groups, type of stay, severity, sex, age and length of stay. The characteristics of hospitalisations were presented using median and range (or mean and SD) for continuous variables and frequencies and proportions for categorical variables. The methods were compared using Student or Wilcoxon rank test for continuous variables and Chi-square or Fisher test for categorical ones. Statistical bilateral significance threshold was set at 5%. Statistical analyses were performed using SAS version 9.1 (SAS Institute, Cary, North Carolina).

## Results

### PAH standardised rates in 2012

**Figure 1:**
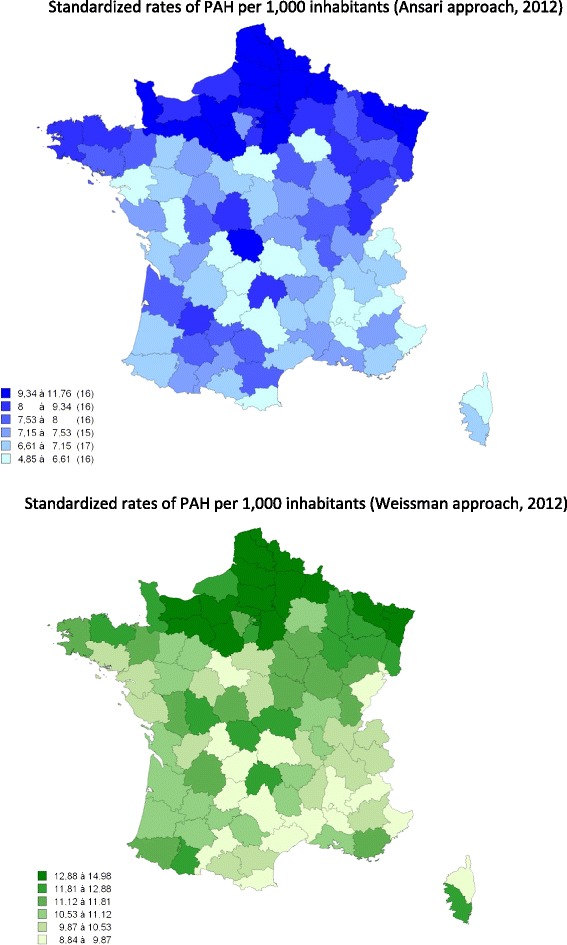
**Maps of standardised rate of PAH in French departments, 2012.**

The total number of discharges in 2012 in France was 26,656,833. The number and proportion of PAH estimated by the Weissman et al. and Ansari et al. approaches were, respectively, 742,474 (2.8%) and 510,206 (1.9%) (Table 3 and Figure 1). Overall, 334,745 discharges were identified by both approaches. The standardised rate of PAH estimated by the Weissman and Ansari approaches were, respectively, 13.3 and 9.0 cases per 1,000 inhabitants.

**Table 3:** **Number of PAH in France in 2012 according to severity, type of event and diagnosis-related groups**

		**Ansari approach**	**Weissman approach**	**p**
		**N (%)**	**N (%)**	
Diagnosis	1 (nervous system)	6,724 (1.3%)	455 (0.1%)	<.001
group	2 (eye)	106 (0.0%)	NA	.
	3 (ENT)	956 (0.2%)	5,262 (0.7%)	.
	4 (respiratory system)	211,490 (41.5%)	229,314 (30.9%)	.
	5 (circulatory system)	252,883 (49.6%)	288,396 (38.8%)	.
	6 (digestive system)	4,671 (0.9%)	50,947 (6.9%)	.
	7 (liver and pancreas)	1,927 (0.4%)	NA	.
	8 (musculoskeletal system)	3,423 (0.7%)	NA	.
	9 (skin)	6,568 (1.3%)	65,787 (8.9%)	.
	10 (endocrine and nutritional)	2,612 (0.5%)	20,807 (2.8%)	.
	11 (urinary system)	3,786 (0.7%)	79,428 (10.7%)	.
	12 (genital, male)	331 (0.1%)	4 (0.0%)	.
	13 (genital, female)	165 (0.0%)	NA	.
	14 (obstetrics)	407 (0.1%)	NA	.
	15 (childbirth)	143 (0.0%)	95 (0.0%)	.
	16 (haematology)	2,607 (0.5%)	NA	.
	17 (haematology, others)	1,319 (0.3%)	NA	.
	18 (infectious diseases)	2,125 (0.4%)	301 (0.0%)	.
	19 (psychiatry)	1,147 (0.2%)	NA	.
	20 (psychiatry, organic)	329 (0.1%)	NA	.
	21 (external causes)	754 (0.2%)	NA	.
	22 (burns)	142 (0.0%)	NA	.
	23 (other)	3,092 (0.6%)	NA	
	25 (HIV)	1,040 (0.2%)	1,521 (0.2%)	.
	26 (polytrauma)	431 (0.1%)	NA	
	27 (transplantations)	415 (0.1%)	157 (0.0%)	
	28 (very short stays)	613 (0.1%)	0	
Type	Surgical procedure	16,002 (3.1%)	98,545 (13.3%)	<.001
	Non-surgical procedure	24,753 (4.9%)	50,899 (6.9%)	.
	Medical stay	466,249 (91.4%)	592,961 (79.9%)	.
	Short stays	3,202 (0.6%)	69 (0.0%)	
Severity	1 (low)	107,578 (21.1%)	229,912 (31.0%)	<.001
	2	150,140 (29.4%)	164,501 (22.2%)	.
	3	143,854 (28.2%)	174,614 (23.5%)	.
	4 (high)	34,814 (6.8%)	37,813 (5.1%)	
Sex	Male	269,771 (52.9%)	370,053 (49.8%)	<.001
	Female	240,435 (47.1%)	372,421 (50.16%)	.
Age (yrs)	(mean, SD)	72 (20)	61 (29)	<.001
	0-17 yr	21,142 (4.1%)	100,629 (13.6%)	<.001
	18-64 yr	111,164 (21.8%)	214,170 (28.9%)	
	65-74 yr	78,589 (15.4%)	90,402 (12.2%)	
	≥75. yr	299,311 (58.7%)	337,273 (45.4%)	
Length of stay (days)	(mean, SD)	9.6 (11.5)	7.3 (8.5)	<.001

**Figure 2:**
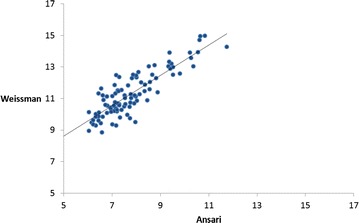
**Correlation between standardised rates of PAH in France in 2012.** Legend: Figure 2 shows the correlation between age and sex-standardised rates of PAH measured by the Weissman and Ansari approaches. Each dot represents a department.

The characteristics of PAH are given in Table 3. There was a significant correlation between diagnoses made by the two approaches (Figure 2), and the two most common causes of PAH (cardiovascular and respiratory systems) are in the same ranking order. However, the Weissman et al. approach identified a lower proportion of respiratory and circulatory system conditions within PAH (38.9 and 30.9% for Weissman and 49.6 and 41.5% for Ansari), and a higher proportion of skin and urinary system conditions (8.9% and 10.7% for Weissman and 1.3 and 0.7% for Ansari). Although the highest percentage of PAH was found in subjects over 75 years of age, the Weissman et al. approach identified significantly older patients. The Weissman et al. approach detects significantly shorter stays (7.3 ± 8.5 days vs. 9.6 ± 11.5 days), with a lower severity level (p < 0.001) and a lower proportion of medical stays (p < 0.01).

At the department level, the standardized rate of PAH ranged from 4.9 to 11.8 cases per 1,000 inhabitants using the Ansari method and from 8.8 to 15.0 cases per 1,000 inhabitants using the Weissman method. Figure 1 suggests an overall North–south gradient, but there are significant differences between the two approaches.

## Discussion

### Overall results

The estimation of PAH according to the Weissman et al. and Ansari et al. approaches ranged from 2.8% of total discharges to 1.9%. The standardised rate of PAH estimated by the Weissman and Ansari approaches were, respectively, 13.3 and 9.0 cases per 1,000 inhabitants. These results are consistent with the recent work of Gusmano et al. that estimated standardized PAH rates between 9.1 and 11.1 per 1,000 inhabitants.

There are significant disparities by conditions groups, age, length of stay, severity level, and proportion of medical stays between the methods.

### Strengths and limitations

This study is based on the nation-wide, all-payers, and public and private hospitals discharge database. Hence it can be considered as exhaustive and representative. By definition, DRGs classify cases according to principal and secondary diagnoses, patient age and sex, the presence of co-morbidities and complications as well as the procedures performed. The PMSI is based on DRGs allowing an exhaustive patient case classification system (i.e. the system of diagnosis-related groupings). The PMSI is standardized and exhaustive but there are some limitations. Inconsistency may occur due to variability in coding in different health institutions because of ignorance or misinterpretation of coding rules. Since 2009, army hospitals have been included, and the definition of primary diagnosis has changed. The primary diagnosis is “the health problem which motivated the admission of the patient, determined at the end of the stay”.

In general, hospital discharges in European countries tend to be related to the number of hospital beds in the country [44]. Trends in hospital discharges may reflect other independent causes. Healthcare demand grows as population’s age. In the present study, PAH in subjects over 65 years of age represent 67 to 72% according to the algorithm. Changes in medical technologies and medical practices are important.

The coding approaches may not be completely relevant for the French population. Weissman et al. are still considered as the gold standard, even though many NCDs (e.g. COPD) are not included. This is why we attempted to compare results with a newer coding system used in the Australian population which is close to the French population but not completely identical. Moreover, some codes are missing (e.g. J82, eosinophilic asthma). A new study is favoured using an instrument specifically targeted to the French population.

### Generalizability

The discrepancies between the two coding approaches are substantial. They may be, at least partly, explained by various factors. Firstly, the two algorithms encompass different disease categories. For instance, Weissman et al. [5] take into account hypokalaemia and infectious diseases which are two very common conditions. Secondly, even when disease categories are labelled in the same way, Ansari et al. [23] exclude a significant proportion of hospitalisations as compared to Weissman et al., due to a more restricted list of ICD codes, through the exclusion of hospitalizations with surgical procedures. Hence, though both methods include hypertension, Ansari et al. coding [23] has fewer ICD-10 codes (2 vs. 15). The Ansari method [23] is consequently more restrictive than the Weissman method [5]. The restricted definition of the Ansari method makes it closer to being “ambulatory care sensitive” compared to the use of the broader definitions of Weissman. However, the study was aimed at finding differences and not at assessing the method of choice.

The results of the study using the Ansari et al. approach [23] are in line with data from many European countries, with the highest rates for cardiovascular and respiratory disorders. However, the rates vary widely depending on the classification and coding methods.

In the Ansari et al. approach, respiratory diseases include communicable and non-communicable diseases (asthma and COPD). The development of a coding exercise is required before these two diseases are studied in the French population.

Large differences exist between the 97 French *Départements*. These may be due to variations in epidemiological patterns, in coding practices, in medical practice and in healthcare supply.

From a policy perspective, our study shows striking differences between two published definitions of PAH. On the one hand, some scholars have advocated that each country develops its own method tailored to the purpose [45]. On the other hand, implementing different methods might prevent useful international comparisons. Nevertheless our study does not allow recommending one method over the other.

## Conclusion

There are significant differences between the Weissman and Ansari methods with reference to potentially avoidable hospitalizations in France in 2012. The method used to measure potentially avoidable hospitalizations is critical, and might influence the assessment of accessibility and performance of primary care.
